# Standardized lung function reference values in rats for translational respiratory research

**DOI:** 10.1038/s42003-026-10123-0

**Published:** 2026-04-24

**Authors:** Gergely H. Fodor, Ferenc Rárosi, Krisztina Boda, Fruzsina Farkas, Fruzsina Kun-Szabó, Álmos Schranc, Petra Somogyi, Ferenc Peták

**Affiliations:** 1 Department of Medical Physics and Medical Informatics, University of Szeged, Szeged, Hungary; 2 Institute of Anesthesiology and Perioperative Medicine, University Hospital Zurich, University of Zurich, Zurich, Switzerland; 3 Cerebral Blood Flow and Metabolism Research Group, Hungarian Centre of Excellence for Molecular Medicine – University of Szeged, Szeged, Hungary; 4 Department of Cell Biology and Molecular Medicine, University of Szeged, Szeged, Hungary

**Keywords:** Translational research, Respiration

## Abstract

Preclinical models are essential for understanding respiratory physiology and disease, but their translational impact is limited by the absence of standardized reference frameworks. Unlike clinical practice, where lung function is interpreted using prediction equations and z-scores, preclinical studies report absolute values, hampering comparability. We established reference equations for airway resistance, tissue damping, tissue elastance, and end-expiratory lung volume in 182 healthy Sprague Dawley and Wistar rats. Using generalized additive models for location, scale, and shape (GAMLSS), we modeled mean and variability as functions of body mass, sex, strain, and positive end-expiratory pressure. The models showed excellent fit and cross-validation, enabling accurate z-score derivation at the individual level. Sex and strain significantly influenced outcomes. Open-source calculators support direct integration into workflows. This framework introduces clinical-style reference equations into preclinical respiratory research, enhancing reproducibility and aligning animal measurements with clinical standards, with implications for computational models, study design, and translational research.

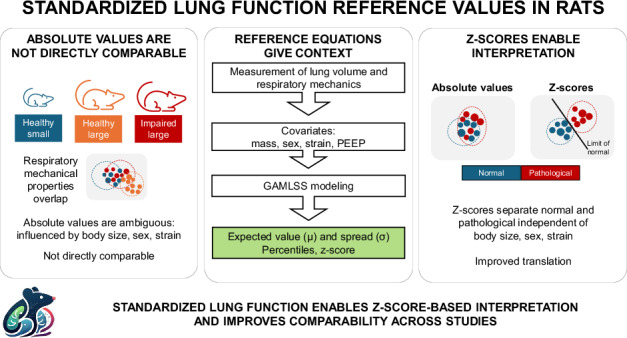

## Abstract (web summary)

Clinical-style reference equations for rat lung function enable z-score-based interpretation of respiratory mechanics, improving standardization, reproducibility, and translational alignment in preclinical respiratory research.

## Introduction

Preclinical animal models are essential for understanding respiratory pathophysiology and evaluating new therapies, but their translational value is often limited by variability in measurement and reporting. Without standardized frameworks, absolute values cannot be reliably compared across experiments, and meaningful differences between groups may be obscured by biological variability^1^. This lack of context hampers reproducibility across laboratories and weakens the alignment between preclinical and clinical research.

Clinical respiratory research has long addressed these issues by interpreting lung function through reference equations and z-scores^2–4^, which place individual measurements in the context of expected biological variability^5,6^. These prediction systems are central to reproducible diagnosis and interpretation in human studies^5,7–11^, yet no equivalent framework exists for preclinical respiratory physiology. Bridging this methodological gap would provide a standardized way to interpret experimental data, improve reproducibility, and enhance the translational relevance of animal models.

The need for such a framework is amplified by the increasing use of advanced systems such as the flexiVent FX (SCIREQ Inc., Montréal, Canada)^12,13^, which enable high-precision measurements of airway and tissue mechanics with high translational value, requiring minimal specialized expertise. While these tools generate large datasets across many laboratories, their full potential remains underexploited in the absence of standardized reference frameworks.

Here we establish clinical-style reference values and prediction equations for key oscillometric respiratory mechanical parameters and end-expiratory lung volume in the two most commonly used strains of healthy rats (Sprague Dawley and Wistar), accounting for body mass, sex, and strain. By modeling both central tendencies and variance with the Generalized Additive Models for Location, Scale and Shape (GAMLSS) framework, the equations enable z-score–based interpretation of individual measurements. We provide open-access tools to facilitate immediate use. Our goal is to deliver a foundational, readily implementable tool that improves interpretability, reproducibility, data quality, and translational relevance in experimental respiratory research, while offering a generalizable strategy for harmonizing preclinical and clinical data.

## Results

We established reference equations for airway resistance (Raw), tissue damping (G), tissue elastance (H), and end-expiratory lung volume (EELV) in 182 healthy Sprague Dawley and Wistar rats.

### Characteristics of measured data

**Fig. 1:**
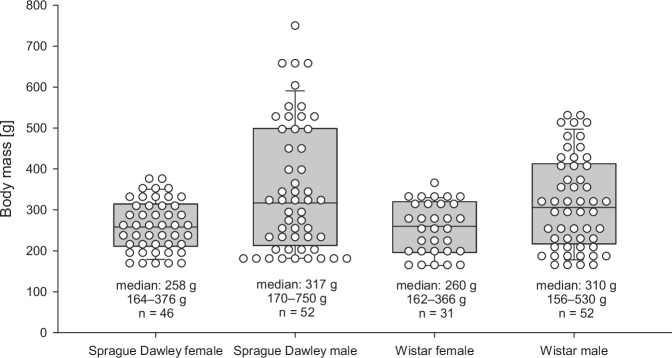
Distribution of body masses within the groups as box plots with overlaid dot-density representation. Boxes represent median and interquartile range; whiskers indicate the 10th and 90th percentiles; dots represent individual observations, arranged in a dot-density format with values binned for clarity. All statistical analyses were performed on the original (unbinned) data.

Distributions of body mass by strain and sex are shown in Fig. 1. Male rats exhibited a wider mass range, reflecting their greater growth potential, while females did not reach higher body mass.

**Fig. 2:**
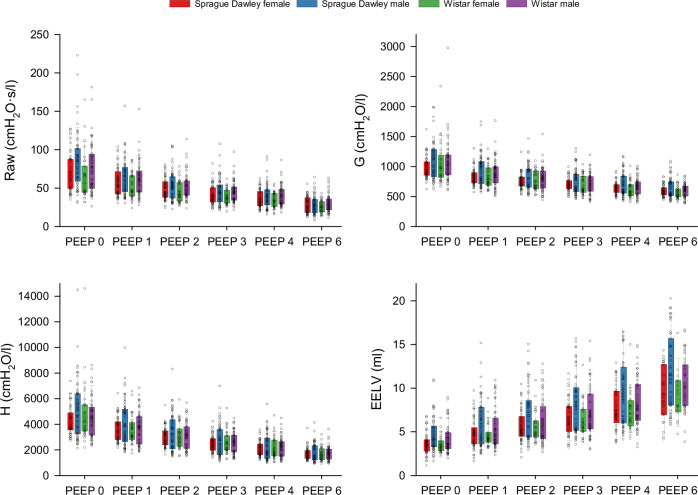
Descriptive statistics of the outcome variables grouped by strain, sex, and PEEP. Raw: airway resistance, G: respiratory tissue damping, H: respiratory tissue elastance, EELV: end-expiratory lung volume. *n* = 46 for Sprague Dawley female, *n* = 52 for Sprague Dawley male, *n* = 31 for Wistar female, *n* = 52 for Wistar male. Boxes represent median and interquartile range; whiskers indicate the 10th and 90th percentiles; individual data points are shown.

Descriptive statistics of outcome variables are presented in Fig. 2 and Supplementary Table 1, grouped by strain, sex, and positive end-expiratory pressure (PEEP). As expected, increasing PEEP reduced Raw, G, and H while increasing EELV. The variability of ranges within each outcome parameter indicated heteroscedasticity, justifying the more complex modeling approach.

### Model fit and validation

Final model summaries are presented in Table 1. Log-transformation was carried out for Raw, G, and H, but not for EELV. Fits were good across outcomes, with *R*^2^ ranging from 0.672 (Raw) to 0.848 (H). Residual analyses (Supplementary Figs. 1–4) showed no systematic trends in the fitted versus residual plots, with residuals symmetrically distributed and near-linear quantile–quantile (Q–Q) plots with deviations only beyond the ±2–3 standard deviation range, indicating adequate variance modeling.

**Table 1:** Model fit and residual diagnostics for the final GAMLSS models function in the *gamlss* package

	Airway resistance (Raw)	Tissue damping (G)	Tissue elastance (H)	End-expiratory lung volume (EELV)
**Link function for µ**	log	log	log	identity
**Link function for σ**	log	log	log	log
***R***^2^	0.672	0.778	0.848	0.837
**Residuals mean**	0.003	<0.001	0.002	0.141
**Residuals standard deviation**	0.272	0.139	0.189	1.692
**Residuals skewness**	0.193	0.713	0.515	0.207
**Residuals excess kurtosis**	0.216	1.838	1.058	1.317

Residual values are calculated as the difference between observed and fitted values. *R*^2^ values are calculated following Cox and Snell. µ: the mean or median of the fitted normal distribution (predicted 50th percentile value). σ: the standard deviation of the distribution.

Model coefficients for each outcome variable are presented in Table 2, with example calculations and full outputs provided in the Online Supplement. Effect sizes are displayed in Supplementary Table 2. Standardized effect sizes indicated that sex effects were consistently large across all outcome parameters, whereas strain effects were small to moderate.

**Table 2:** Coefficients for the final GAMLSS models

	Coefficients	Airway resistance (Raw) [cmH_2_O ∙ s/l]	Tissue damping (G) [cmH_2_O/l]	Tissue elastance (H) [cmH_2_O/l]	End-expiratory lung volume (EELV) [ml]
**Coefficients for predicted value (µ)**	**a0 (intercept)**	5.36400	7.87594	9.98623	−5.29284
	**a1 (sqrt(mass [g]))**	−0.07261	−0.06022	−0.09948	0.58809
	**a2 (strain)**	−0.07227	−0.05717	−0.05654	−0.16721
	**a3 (sex)**	0.24217	0.16888	0.20138	−0.27947
	**PEEP 0 cmH**_**2**_**O**	0.00000	0.00000	0.00000	0.00000
	**PEEP 1 cmH**_**2**_**O**	−0.20376	−0.17255	−0.20777	0.82506
	**PEEP 2 cmH**_**2**_**O**	−0.36403	−0.27962	−0.39299	1.57536
	**PEEP 3 cmH**_**2**_**O**	−0.51259	−0.36856	−0.58629	2.47960
	**PEEP 4 cmH**_**2**_**O**	−0.65403	−0.45209	−0.76092	3.46782
	**PEEP 6 cmH**_**2**_**O**	−0.96527	−0.53716	−0.98447	5.32320
**Coefficients for standard deviation (σ)**	**b0 (intercept)**	−1.17594	−1.85751	−1.89371	−0.87845
	**b1 (mass [g])**	—	—	0.00078	0.00413
	**b2 (strain)**	−0.12428	—	—	−0.13945
	**b3 (sex)**	—	0.12800	−0.07682	−0.41105
	**PEEP 0 cmH**_**2**_**O**	0.00000	0.00000	—	0.00000
	**PEEP 1 cmH**_**2**_**O**	−0.19736	−0.25486	—	0.02265
	**PEEP 2 cmH**_**2**_**O**	−0.21221	−0.29300	—	0.13399
	**PEEP 3 cmH**_**2**_**O**	−0.16229	−0.23964	—	0.27625
	**PEEP 4 cmH**_**2**_**O**	−0.05226	−0.26472	—	0.45725
	**PEEP 6 cmH**_**2**_**O**	0.06301	−0.32089	—	0.70147

The mean of the distribution (µ) can be calculated as:

$$\mu ={a}_{0}+{a}_{1}\cdot \sqrt{{mass}\,[g]}+{a}_{2}\cdot {I}_{{Wistar}}+{a}_{3}\cdot {I}_{{male}}+{PEEP}{{\_}}{effect}$$

Predicted values are obtained by exponentiating µ for Raw, G, and H, while EELV uses µ directly.

The standard deviation (σ) is calculated as:

$$\sigma ={exp} \left({b}_{0}+{b}_{1}\cdot {mass}\,[g]+{b}_{2}\cdot {I}_{{Wistar}}+{b}_{3}\cdot {I}_{{male}}+{PEEP}{{\_}}{effect}\right)$$

Indicator variables (*I*) are defined as: *I*_Wistar_ = 1 for Wistar and 0 for Sprague Dawley; *I*_male_ = 1 for males and 0 for females. “PEEP effect” refers to the coefficient corresponding to the applied PEEP level. Body mass should be used in grams. Parameters marked as ‘—’ were excluded from the final model.

Full summaries, including standard errors and *p*-values, are provided in the Online Supplement.

All included predictors were highly significant. Some PEEP levels were found to be non-significant when considering σ fits for Raw and EELV; however, the overall effect of PEEP was significant, and its omission would have been detrimental to model quality. Similarly, when considering the standard deviation (σ) submodel for H, the effect of sex was found to be significant only at a 10% level (*p* = 0.078), but its omission worsened the fit and generalizability of the model.

Residual diagnostics demonstrated a close fit to the normal distribution across all models with only minor deviations (Supplementary Figs 1–4). This confirms that both the mean (µ) and standard deviation (σ) submodels provide satisfactory fits to the data, including heteroscedasticity present in the outcome variables. These results validate the models’ use for both prediction and z-score-based interpretation at the individual level.

### Cross-validation performance

**Fig. 3:**
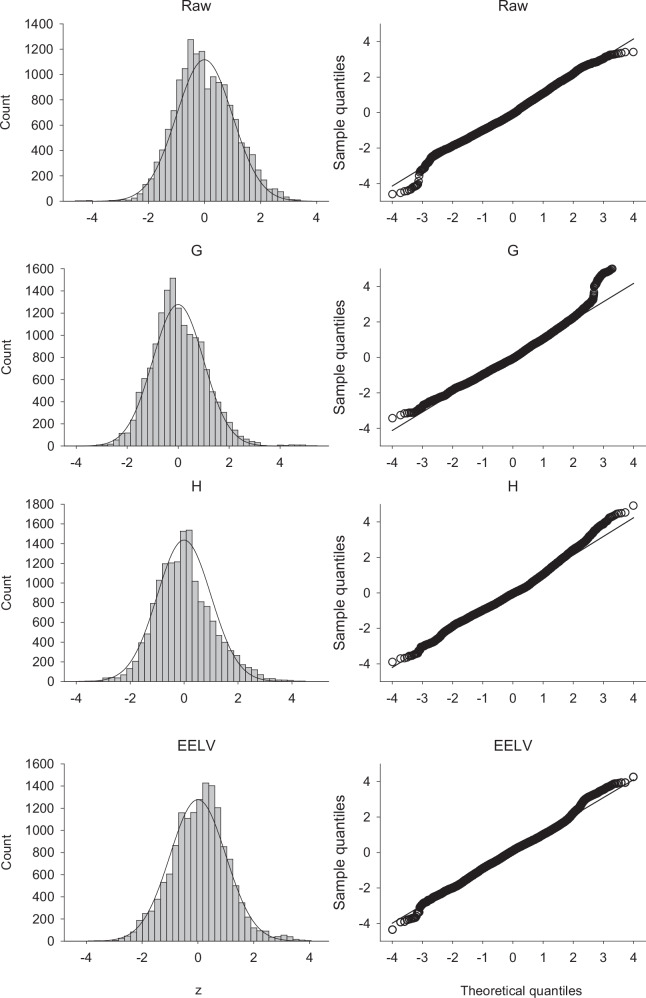
Distribution of the z-scores obtained during the 50-fold Monte Carlo cross-validation. Left panels show the histograms of the z-scores with normal distribution for reference (continuous line). The right panels show Q–Q plots with trend lines indicated. Raw: airway resistance, G: tissue damping, H: tissue elastance, EELV: end-expiratory lung volume.

Monte Carlo cross-validation (50-fold) supported robust predictive performance (Table 3, Fig. 3). Z-scores were symmetrically distributed with means near zero and standard deviations close to one. Coverage for 90% and 95% intervals closely matched theoretical expectations: approximately 90% of observations fell within ± 1.645 and approximately 95% within ±1.96, with only minor deviations. Root mean square error (RMSE) values were markedly lower than the overall variability (expressed as standard deviation) of the outcomes (25.010 for Raw, 253.048 for G, 1546.229 for H, 3.365 for EELV), confirming the models’ ability to capture central tendencies and provide meaningful z-scores for individual animals.

**Table 3:** Results of the Monte Carlo cross-validation of the models

	Airway resistance (Raw)	Tissue damping (G)	Tissue elastance (H)	End-expiratory lung volume (EELV)
**z-score mean (standard deviation)**	0.006 (1.040)	0.025 (1.044)	0.015 (1.063)	0.062 (1.014)
**z-score range**	−4.609 to 3.407	−3.431 to 5.436	−3.900 to 4.918	−4.359 to 4.256
**90% coverage**	88.7%	89.6%	88.4%	89.8%
**95% coverage**	94.4%	94.4%	93.2%	94.3%
**RMSE mean (standard deviation)**	15.189 (1.651)	124.173 (18.838)	603.979 (57.235)	1.709 (0.141)
**RMSE range**	11.280–19.179	99.117–165.349	460.121–706.366	1.330–1.997
**MAE mean (standard deviation)**	10.902 (0.987)	87.547 (6.604)	434.128 (37.826)	1.241 (0.110)
**MAE range**	8.705–13.080	73.971–99.807	344.377–511.858	0.959–1.521

Values are averaged over 50 iterations using a 70–30 random train-test split. RMSE: root mean squared error, defined as the square root of the mean of squared differences between measured and predicted values. MAE: mean absolute error, defined as the mean of absolute differences between measured and predicted values. Coverage values refer to the proportion of z-scores within ±1.645 (corresponding to the central 90% interval, between 5th and 95th percentiles of the reference distribution) and ±1.96 (corresponding to the central 95% interval, between 2.5th and 97.5th percentiles of the reference distribution) in the validation data.

### In vivo validation of the models

To illustrate practical application, we applied the reference equations to an independent dataset from an ongoing study, in which Sprague Dawley rats of both sexes were treated with bleomycin to induce lung fibrosis and then measured at PEEP 6. While both sexes underwent the same procedure, absolute values of H showed a clear separation between male and female animals with considerable overlap between control and bleomycin-treated animals, making direct comparison cumbersome, partly due to differences in body mass. In contrast, z-score interpretation removed this separation and also highlighted that the majority of bleomycin-treated animals exhibited H values above the expected normal range (i.e. above the 95th percentile, corresponding to *z* > + 1.645 under the reference distribution), whereas all controls except one animal remained within the healthy range (Supplementary Fig. 5). This example illustrates the framework’s potential for in vivo validation and its utility in preclinical experimental design.

## Discussion

The present study provides the first reference equations for airway and respiratory tissue mechanical parameters and end-expiratory lung volume in anesthetized ventilated rats, establishing a standardized framework for reporting preclinical lung function according to clinical standards.

By using the GAMLSS framework, both the expected (mean) values and the associated variability (standard deviation) can be modeled for each outcome parameter as a function of body size, sex, strain, and PEEP. As such, this framework enables the calculation of standardized z-scores for individual measurements. This approach parallels reference systems used in human respiratory physiology^5^ and supports consistent interpretation of experimental data across studies and groups. The strong performance of the models across strains, sexes, and PEEP levels validates their use for reproducible interpretation of preclinical data. The near linearity of the Q–Q plots indicates an overall good fit, with the minor tail deviations being a sign of biological variability. Importantly, the framework does not simply refine existing reporting but introduces a shift toward standardized, context-aware assessment in animal physiology, akin to clinical standards.

Physiologically, our findings also shed light on strain and sex dependence of respiratory mechanics in rats, with significant effects of both covariates on all outcome parameters, even when accounting for body size. This is analogous to findings in humans, where both sex and ethnicity have been found as significant predictors of lung function and mechanics^5,14^. It is noteworthy, however, that not only is the magnitude of the outcome parameters affected by sex and strain, but the variability of all parameters also shows significant sex and/or strain dependence. More broadly, this demonstrates the necessity of accounting for biological variability in preclinical research, rather than relying on absolute measurements. An interesting finding is that effect sizes for strain are consistently smaller in magnitude than those associated with sex. The small-to-moderate strain-related effects, corresponding to approximately 4–7% differences across parameters, suggest that while strain remains a significant predictor of lung function, extrapolation of these findings may be feasible with only modest loss of accuracy. This interpretation should nevertheless be confirmed in future studies, including a broader range of strains.

The forced oscillatory technique is commonly used both in animal models^15,16^ and humans^17^ as an alternative to spirometry measurements, to characterize respiratory mechanics, with similar reference value frameworks existing for various human populations^11,14^, therefore having a great translational relevance. The present equations were also derived using forced oscillations to measure respiratory mechanics and plethysmography in anesthetized rats, but the framework is not limited to a single system. Equivalent devices, such as the widely used flexiVent^12,13,18^, yield comparable input impedances and constant-phase model parameters, and can therefore adopt these equations directly, consistent with prior evidence of accuracy within 1% against known test loads^19^. In small-rodent studies, plethysmography is the most common method for measuring EELV, including in the present work. Other approaches for respiratory mechanics, such as multiple linear regression^20–22^, transfer impedance^23,24^, and for lung volumes, such as inert gas washout^25^ or imaging^26^ have also been applied in rodents. Detailed methodological considerations for these techniques are provided in Supplementary Information (as part of Supplementary Notes). Thus, while the concept of clinical-style reference modeling is broadly generalizable, the numerical coefficients remain specific to the measurement conditions.

Although the present study focuses on in vivo whole-lung measurements and statistical reference modeling rather than regional or mechanistic simulations, it aligns with a broader translational pipeline in which computational frameworks integrate regional lung behavior with organ-level function. Multiscale and inverse modeling approaches typically aim to reproduce or constrain global observables such as respiratory system impedance and lung volume, derived from forced oscillatory measurements or related techniques^1,27–29^ In this context, standardized reference equations for Raw, G, H, and EELV provide essential normative targets, enabling computational and imaging-based studies to distinguish physiological variability from pathological deviation and to improve cross-study comparability.

Beyond respiratory mechanics, standardized physiological reference frameworks in animal research remain relatively uncommon. In some systems, such as rat cardiac function, normative echocardiographic datasets have been reported, typically as summary reference ranges rather than formal multi-covariate reference equations^30^. Similar reference interval-based approaches exist for hematologic and biochemical parameters in rats^31,32^, mirroring how these physiological indices are commonly reported in human populations. In contrast, respiratory function in humans is routinely interpreted using multivariable reference equations accounting for key biological covariates. The present framework highlights how multivariable reference modeling, which is already central to clinical interpretation, can be implemented in preclinical settings, while also illustrating the conditions under which such approaches may be relevant for other physiological systems from a translational perspective.

Several limitations should be acknowledged. Respiratory mechanical indices reflect combined lung and chest wall contributions^33,34^, which may lead to underestimation of lung-specific changes, particularly in smaller animals or at higher PEEP. Although this is the standard approach and is closely analogous to measurements made in humans, and therefore does not constitute a major limitation, the relative contribution of the lung is not fully explored across the entire range of body masses examined in the present study. This aspect, therefore, warrants some consideration. In general, the contribution of the lung to G is approximately 45–65% and approximately 60–70% to H, depending on PEEP^25^. Measurements of respiratory mechanics in rodents require general anesthesia, which may influence respiratory properties; however, our prior work showed no major differences in respiratory mechanics and lung volumes across commonly used anesthetic regimens^35^. While repeated measurements might suggest a random-effects approach, incorporating subject-level effects reduced predictive accuracy in cross-validation (a middle 90% and 95% coverage of 61.3% and 70.0%, respectively, for Raw using random effects, compared with 88.7% and 94.4%, respectively, without) and was therefore not adopted. The present equations apply to Sprague Dawley and Wistar rats within defined body mass ranges (approximately 160–370 g for females and 160–530 g for males, extending to 750 g in male Sprague Dawley), and extrapolation beyond these ranges should be made with caution. As strain was a significant predictor, the models are most accurate for Sprague Dawley and Wistar rats, though limited applicability to related strains may still be expected^36^. Finally, while the framework was supported by validation in an independent experimental cohort, all data were acquired within a single laboratory; external validation across independent laboratories would be beneficial to further assess the generalizability of the proposed reference framework.

As illustrated in the bleomycin validation example, z-scores can ease direct comparison of diverse experimental groups, where differences due to body mass and sex can mask treatment effects when considering only absolute values, demonstrating how this framework can aid in interpreting preclinical interventions against the background of expected biological variability.

This framework introduces clinical-style reference equations and z-score interpretation into preclinical respiratory research, providing a standardized way to determine whether observed changes exceed expected biological variability. Aligning animal measurements with the interpretive standards used in clinical practice, it enhances reproducibility, improves data quality through more rigorous reporting, and facilitates comparability across laboratories, thereby narrowing the translational gap between experimental models and human disease.

To support practical adoption, we provide open-access R models and an Excel calculator, both compatible with widely used statistical workflows. Although developed in rats, the approach can be extended to other species and adapted to additional physiological systems, enabling the creation of harmonized or even cross-species reference models.

Beyond methodological value, standardized reference equations may also strengthen preclinical research by allowing effect sizes to be interpreted relative to expected biological variability rather than solely against cohort-specific controls. This variability-aware approach mirrors clinical pulmonary function testing, where deviation from predicted normal values is routinely used to contextualize disease severity. This is exemplified by the bleomycin-induced pulmonary fibrosis validation, where z-score–based interpretation of tissue elastance enabled clear discrimination between diseased and control animals across sexes, highlighting the biological utility of the reference framework. Although constant-phase model parameters are not typically obtained in humans, oscillometric indices sample airway and tissue mechanical domains that correspond to the mechanical domains represented in the model. Consequently, deviations of tissue damping and elastance from their reference distributions in experimental models may be interpreted in relation to clinically familiar patterns of peripheral airway involvement, supporting careful conceptual translation without assuming parameter equivalence^37–39^

In summary, the framework advances preclinical respiratory research by enabling z-score-based interpretation of lung function in rats and, with extension to other models and systems, offers a scalable tool for harmonizing preclinical findings with clinically established principles of lung function interpretation and narrowing the translational gap between experimental models and human disease.

## Materials and methods

### Ethical approval

This experimental protocol was approved by the National Food Chain Safety and Animal Health Directorate of Csongrád-Csanád County, Hungary (no. XXXII./2110/2019) on 16 December 2019. We have complied with all relevant ethical regulations for animal use, specifically the guidelines of the Scientific Committee of Animal Experimentation of the Hungarian Academy of Sciences (updated Law and Regulations on Animal Protection: 40/2013 [II. 14.], the Government of Hungary). All procedures are reported in compliance with the ARRIVE guidelines.

### Experimental animals

Healthy Sprague Dawley (CD® IGS Rat, Charles River, Germany) and Wistar (Wistar IGS Rat, Charles River, Germany) rats of both sexes were studied. Of 200 rats, 18 were excluded from the analysis due to respiratory infection (3), premature loss (9), or incomplete data (6). After all the exclusions, we included 52 male Sprague Dawley (median: 317 g, min-max: 170–750 g), 46 female Sprague Dawley (258 g, 164–376 g), 53 male Wistar (310 g, 156–530 g), and 31 female Wistar rats (260 g, 162–366 g) in the final analyses. Male and female animals were kept in the same room in separate cages.

### Animal preparations

Anesthesia was induced with intraperitoneal pentobarbital sodium (45 mg/kg)^35,40^. Tracheostomy was performed after local infiltration with lidocaine (2–4 mg/kg). Based on body size, a metal cannula of either 2.0-mm outer diameter or 2.5-mm outer diameter (Harvard Apparatus, South Natick, MA, United States) was used. All measurements were corrected for the applied cannula.

Mechanical ventilation was performed using a rodent ventilator (Model 683, Harvard Apparatus, South Natick, MA, United States) with room air at a tidal volume of 10 ml/kg using a frequency (60–70 BPM) that achieved normocapnia at a PEEP of 2 cmH_2_O. The left femoral vein was cannulated for anesthesia maintenance. Anesthesia was maintained using continuous intravenous infusions of propofol (30 mg/kg/h) and fentanyl (5 μg/kg/h)^35,40^. The left femoral artery was prepared for blood gas sampling and blood pressure measurement. The arterial line was connected to a physiology data collection system (Powerlab 8/35 and Labchart 8, ADInstruments, Dunedin, New Zealand) using a pressure transducer (MLT0380 Reusable blood pressure Transducer, ADInstruments, Dunedin, New Zealand).

Neuromuscular blockade (pipecuronium bromide, 0.1 mg/kg, every 30 min) was used during respiratory mechanical measurements. Body temperature was maintained constant at 37.0 °C ± 0.5 °C with a heating pad and rectal temperature probe. Arterial blood gases were periodically sampled to ensure normocapnia and normoxia. ECG, blood pressure, and airway pressure were continuously monitored. Animals were humanely terminated and excluded from analysis if signs of significant physiological instability or illness were observed, including evidence of respiratory infection, sustained hemodynamic instability, abnormal gas exchange, or other clinical deterioration incompatible with the assessment of normal respiratory mechanics.

### Measurement of EELV

EELV was measured with a custom whole-body plethysmograph (1.91 L), as described previously. Briefly, the anesthetized animal was placed in the sealed Plexiglas box, and identical differential pressure transducers (24PCEFA6D, Honeywell, Charlotte, NC, USA) were connected to the trachea and to the box. Measurements were performed at increasing PEEP levels of 0, 1, 2, 3, 4, and 6 cmH_2_O, with a recruitment maneuver carried out to standardize lung volume at each PEEP. During measurement, the tracheal cannula was occluded for 10–15 s, and ventilation was stopped at end-expiration, where thoracic gas volume approximates EELV, while the animal made spontaneous breathing efforts against the closed trachea. Pressures inside the box (Pbox) and in the trachea (Ptrach) were measured during this period.

Respiratory efforts during occlusion were identified from the Ptrach signal. A threshold was applied to exclude cardiogenic oscillations, and only data points exceeding this threshold were included in the analysis. The relationship between Pbox and Ptrach was then determined by linear regression of Pbox versus Ptrach during inspiratory efforts. Assuming that pressure changes were small relative to atmospheric pressure, EELV was calculated from the slope of this relationship (*s* = ΔPbox/ΔPtrach), based on the Boyle–Mariotte law^41^:

$${EELV}=-s\beta \left({V}_{{box}}-{V}_{{rat}}\right)-{V}_{{ds}}$$

where β = (Patm – PH_2_O)/Patm corrects for water vapor pressure at body temperature in alveolar gas, Vbox is the chamber volume, Vrat is the volume of the animal (estimated from body mass, using an average body density of 1.025 g/cm^3^, based on reported densities of adipose, lean, and skeletal tissues and typical body composition in rats^42^), and Vds is the instrumental dead space between the tracheal cannula and the occlusion site. In the present setup, Vds was determined as 0.53 ml while using the 2-mm and 0.65 ml while using the 2.5-mm tracheal cannula. Linearity of the Pbox–Ptrach relationship was verified for each maneuver.

In some cases, measurements at the highest PEEP were not possible due to a lack of breathing effort. The scientists performing the analyses were blinded to group allocation.

### Measurement of respiratory mechanics

Respiratory mechanical parameters (Raw, G, H) were measured by the forced oscillation method as described in detail previously^35^. Briefly, ventilation was stopped at end-expiration, and a short (8 s) apnea was initiated. A stopcock was turned to disconnect the ventilator from the breathing circuit and to connect the respiratory system of the animal to the loudspeaker-in-a-box system.

A loudspeaker in a box with the box maintained at the set PEEP level was used to generate a small amplitude (±1 cmH_2_O) pseudorandom forcing signal with 23 non-integer multiple frequency components of a 0.25-Hz base frequency (0.5–20.75 Hz), covering the frequency range relevant for airway and tissue mechanics. Two identical differential pressure transducers (24PCEFA6D, Honeywell, Charlotte, NC, USA) were connected to both sides of a wave tube with known geometric parameters (2-mm internal diameter, 100-cm length).

The pressure signals were digitized at a sampling rate of 256 Hz using an analog-digital data acquisition board (NI USB-6211, National Instruments, Austin, TX). The digitized signals were analyzed in the frequency domain after performing Fast Fourier Transform (FFT) on them (4-s time window, 97% overlapping). The input impedance of the respiratory system (Zrs) was calculated from the transfer function between the lateral pressures measured along the wave tube from the recordings^43^. At least 3 consecutive and consistent recordings (based on spectral coherence and stability of impedance spectra) were obtained at each level of PEEP and ensemble-averaged for analysis. The input impedance of the endotracheal tube and the connections were determined separately using the same setup without the animal, and then subtracted from each recording.

The constant-phase model was fitted to the Zrs spectra using nonlinear least-squares fitting by minimizing the relative difference between the measured and modeled impedance data^37^:

$${Z}_{{rs}}={R}_{{aw}}+i\cdot \omega \cdot {I}_{{aw}}+\frac{G-i\cdot H}{{\omega }^{\alpha }}$$

where *i* is the imaginary unit, *ω* is angular frequency (2*πf*), and *α* is $\frac{2}{\pi }$∙atan(*H/G*).

The model consists of a frequency-independent resistance (Raw) and inertance (Iaw) in series with a constant-phase tissue compartment composed of tissue damping (G) and tissue elastance (H). The scientists performing the analyses were blinded to group allocation.

### Study protocol

After preparation, animals were placed in the plethysmography box, and EELV was measured at increasing PEEP levels of 0, 1, 2, 3, 4, and 6 cmH_2_O. PEEP was then lowered to 2 cmH_2_O, neuromuscular blockade was initiated, and respiratory mechanics were measured using forced oscillations at the same increasing PEEP levels at 0, 1, 2, 3, 4, and 6 cmH_2_O. PEEP sequence was kept identical to ensure standard volume history. Furthermore, at each step, volume history was standardized using recruitment maneuvers by temporarily occluding the expiratory port of the ventilator, resulting in a single breath of double tidal volume. After the application of the recruitment maneuver, data was collected following a 3-min equilibration period. Each experimental step took about 8–10 min.

### Statistics and reproducibility

Statistical analyses were performed using R (version 4.5.1,^44^), with significance set at *p* < 0.05. Visual inspection of histograms revealed that body mass was right-skewed, so its descriptive statistics are reported as medians and ranges (minimum–maximum).

Outcome variables (Raw, G, H, and EELV) were modeled using the GAMLSS framework with the Gaussian normal (NO) distribution. This approach enables simultaneous modeling of the mean (μ) and standard deviation (σ), thereby accommodating heteroscedasticity. Models were fitted using the *gamlss* package (version 5.4–22) of R^45^. Raw, G, and H were log-transformed to address observed skewness and stabilize variance, while EELV was modeled untransformed. Although formal normality tests were not conducted, visual inspection of histograms and residual plots supported the use of log-transformation.

Separate GAMLSS models were built for each outcome. Candidate predictors for μ included the square root of body mass, strain, sex, and PEEP, while σ included body mass, strain, sex, and PEEP. The selection of predictors for the reference equations was guided by principles analogous to those used in clinical lung function reference systems, such as the Global Lung Function Initiative (GLI)^5^ framework in humans. In this context, rat strain serves as a functional analog to ethnicity, sex is included as a biological determinant of lung mechanics, and body mass captures inter-individual differences in body size and growth. Unlike humans, where age and height are modeled separately, age-related changes in lung mechanics in rats are closely coupled to somatic growth; therefore, body mass was used as a pragmatic surrogate capturing both size- and age-related effects. This approach reflects shared modeling principles rather than direct equivalence between species.

Pilot analyses comparing body mass and the square root of body mass found that the square root of body mass resulted in superior model fits. PEEP was modeled as an ordinal variable. Selection of final predictors from the whole candidate pool for each model was not based solely on Generalized Akaike Information Criterion (GAIC) but followed a more comprehensive approach based on incorporating residual diagnostics and cross-validation performance, which considered both model parsimony and predictive generalizability to mitigate overfitting. In this process, additional terms such as covariate interactions were tested but excluded when they did not improve cross-validated performance. This strategy resulted in models that are both robust and easier to interpret in practical settings^46–48^.

The final models can be summarized using the following general structure:

$$\mu = {a}_{0}+{a}_{1}\cdot \sqrt{{mass}}+{a}_{2}\cdot I({{\mbox{strain}}} = {{\mbox{Wistar}}}) +{a}_{3}\cdot I({{\mbox{sex}}} = {{\mbox{male}}})+{{\mbox{PEEP}}}\_{{\mbox{effect}}}$$

$$\sigma = \exp\left(b_{0} + b_{1}\cdot {{\mbox{mass}}} + b_{2}\cdot I({{\mbox{strain}}} = {{\mbox{Wistar}}}) + b_{3}\cdot I({{\mbox{sex}}} = {{\mbox{male}}}) + {{\mbox{PEEP}}}\_{{\mbox{effect}}} \right)$$

Here, a_0_–a_3_ and b_0_–b_3_ are regression coefficients for μ and σ, respectively; and PEEP_effect captures the effects of each PEEP level. Because a log link was applied to σ in all models, σ is modeled on the log scale and therefore calculated by exponentiating the linear predictor, ensuring strictly positive standard deviation values. Depending on the specific model, some terms are missing from particular equations, as shown in the results. When fitting for μ, a logarithmic transformation was carried out for all variables, except for EELV. Therefore, when a log-transformation is in place (for Raw, G, and H), exponentiation of the calculated μ is also necessary to ensure that predicted and measured values are in the same domain.

Model diagnostics included residual plots and Q–Q plots^49^, alongside Monte Carlo cross-validation as the primary evaluation method (50 iterations, 70/30 train-test splits) to assess generalizability. Performance metrics included standardized residuals (z-scores), root mean square error (RMSE, defined as the square root of the mean of squared differences between measured and predicted values), and mean absolute error (MAE, defined as the mean of absolute differences between measured and predicted values). Coverage values were calculated from the proportion of z-scores within ±1.645 (corresponding to the central 90% interval, between 5th and 95th percentiles of the reference distribution) and ±1.96 (corresponding to the central 95% interval, between 2.5th and 97.5th percentiles of the reference distribution) in the validation data. Models were selected based on z-score symmetry and predictive accuracy rather than information criteria alone. Penalized splines were also tested for smoothing body mass, but cross-validation clearly showed that their inclusion resulted in poor generalizability and extreme standardized residuals at the distribution boundaries, despite their favorable GAIC values, suggesting overfitting in that case.

Effect sizes for strain and sex were calculated from the models as percentage changes for the µ and σ submodels and by calculating standardized effect sizes analogous to Cohen’s d for the µ submodel. Effect sizes were classified as *d*(0.2) = small, *d*(0.5) = medium, *d*(0.8) = large, *d*(1.2) = very large^50^.

Further considerations about modeling choices are provided in Supplementary Information.

### Reporting summary

Further information on research design is available in the Nature Portfolio Reporting Summary linked to this article.

## Supplementary information

Supplementary Information

Description of Additional Supplementary Files

Supplementary Data 1

Supplementary Data 2

Reporting Summary

## Data Availability

Additional numeric data supporting the findings of this study are available from an institutional data repository at 10.82570/SZTE_Datarepo_n05z8-1xc41^51^.

## Code availability

An Excel calculator (Supplementary Data 1), compiled R model objects and custom R scripts for computing predicted values, percentiles (5th and 95th), and z-scores (Supplementary Data 2) are provided as Supplementary Data and are also archived at Zenodo (10.5281/zenodo.17055086)^52^. The version associated with this study (v1.0) is available at 10.5281/zenodo.17055087. The corresponding GitHub repository (https://github.com/fodorgergely/reform-rat-lung-function) provides the most recent development version of the code, including scripts for model fitting and application, and full usage documentation. Percentiles are derived from the reference distribution under the assumption of normal residuals (e.g., the 95th percentile corresponds to *z* = +1.645), which showed good agreement with observed data.
