# Supplementary material for: Standardized lung function reference values in rats for translational respiratory research
Source: Commun Biol. 2026 Apr 24;9:626. doi: 10.1038/s42003-026-10123-0 (PMC13156265; doi:10.1038/s42003-026-10123-0)
Supplement: Supplementary file 1 — Supplementary Information [file 42003_2026_10123_MOESM1_ESM.pdf]

STANDARDIZED LUNG FUNCTION REFERENCE VALUES IN RATS FOR TRANSLATIONAL RESPIRATORY RESEARCH

Gergely H. Fodor[1], Ferenc Rárosi[1], Krisztina Boda[1], Fruzsina Farkas[1], Fruzsina Kun-Szabó[1], Álmos Schranc[1,2], Petra Somogyi[1,3,4], Ferenc Peták[1]

ONLINE DATA SUPPLEMENT

[1] Department of Medical Physics and Medical Informatics, University of Szeged, Szeged, Hungary

[2] Institute of Anesthesiology and Perioperative Medicine, University Hospital Zurich, University of Zurich, Zurich, Switzerland

[3] Cerebral Blood Flow and Metabolism Research Group, Hungarian Centre of Excellence for Molecular Medicine – University of Szeged, Szeged, Hungary

[4] Department of Cell Biology and Molecular Medicine, University of Szeged, Szeged, Hungary

Running title: Standardized lung function reference values in rats

SUPPLEMENTARY RESULTS

Practical examples

To facilitate the application of the presented models, let us consider some examples and calculate predictions along with z-score values for measured parameters.

Example 1) Female Sprague Dawley rat, mass of 230 g, measured at a PEEP of 2 cmH_2O. Measured Raw: 41.82 $cmH_2O\cdot s/l$

To calculate the predicted value and z-score for Raw in this animal, one must look up the coefficients from Table 2. To calculate the mean of the distribution, the following equation should be used:

$$\mu = a_0 + a_1 \cdot \sqrt{mass\ [g]} + a_2 \cdot I_{Wistar} + a_3 \cdot I_{male} + PEEP\_effect$$

By substituting the coefficients from the top part of Table 2, one gets the following equation:

$$\mu = 5.364 - 0.07261 \cdot \sqrt{230} - 0.07227 \cdot 0 + 0.024217 \cdot 0 - 0.36403 = 3.898785$$

To get the predicted value of Raw, μ should be exponentiated, that gives 49.34 $cmH_2O\cdot s/l$ as the predicted value. To calculate the z-score, the standard deviation of the distribution should also be calculated, using the formula:

$$\sigma = exp(b_0 + b_1 \cdot mass\ [g] + b_2 \cdot I_{Wistar} + b_3 \cdot I_{male} + PEEP\_effect)$$

By substituting the coefficients from the bottom part of Table 2, we get the following equation:

$$\sigma = exp(-1.17594 - 0.12428 \cdot 0 - 0.21221) = 0.249537$$

Since the model is fitted to log(Raw), to get the z-score, we should use:

$$z = (\log(measured) - \mu)/\ \sigma\ = (\log(41.82) - 3.8988)/\ 0.2495\ \approx (3.73 - 3.90)/0.25$$

This results in $z = -0.663$ (where *log* is the natural logarithm).

Example 2) Male Wistar rat, mass of 170 g, measured at a PEEP of 0 cmH_2O. Measured EELV: 2.00 ml

To calculate the predicted value and z-score for EELV in this animal, one must look up the coefficients from Table 2. To calculate the mean of the distribution, the following equation should be used:

$$\mu = a_0 + a_1 \cdot \sqrt{mass\ [g]} + a_2 \cdot I_{Wistar} + a_3 \cdot I_{male} + PEEP\_effect.$$

By substituting the coefficients from the top part of Table 2, one gets the following equation:

$$\mu = -5.29284 + 0.58809 \cdot \sqrt{170} - 0.16721 \cdot 1 - 0.27947 \cdot 1 + 0 = 1.928235$$

To get the predicted value of EELV, μ should not be exponentiated, that gives 1.928 ml as the predicted value. To calculate the z-score, the standard deviation of the distribution should also be calculated, using the formula:

$$\sigma = exp(b_0 + b_1 \cdot mass\ [g] + b_2 \cdot I_{Wistar} + b_3 \cdot I_{male} + PEEP\_effect)$$

By substituting the coefficients from the bottom part of Table 2, we get the following equation:

$$\sigma = exp(-0.87845 + 0.00413 \cdot 170 - 0.13945 \cdot 1 - 0.41105 \cdot 1 + 0) = 0.483551$$

To get the z-score, we should use

$$z = (measured - predicted)/\ \sigma\ \approx (2.00 - 1.93)/\ 0.484$$

This results in $z = 0.148$. In this case, we do not need to take the logarithm of the measured value, as with no logarithmic transformation was applied to EELV.

In addition to manual calculation from the coefficients, the same results can be obtained for both examples using the Excel calculator in the Supplementary Material (Supplementary Data 1), by filling in cells B4 to B7 with the details of the rat and measurement condition. If z-score values are of interest, the measured values should be entered in cells B10 to B13. The attached R script can also be used to reproduce the same results. It is provided in the Online data supplement as the R script (*.R* file) together with the compiled *.rds* models (Supplementary

Data 2). While the summaries below retain the original factor labels (e.g. ‘female’ and ‘male’) for readability, the published *.rds* model objects are recoded to use numeric levels for categorical predictors to ensure compatibility across environments. The calculators are also available at https://github.com/fodorgergely/reform-rat-lung-function, where the most recent version and documentation can be found.

Supplementary Table 1: Descriptive statistics of the outcome variables grouped by strain, sex, and PEEP. Values given as median (minimum – maximum). Raw: airway resistance [cmH$_2$O·s/l], G: respiratory tissue damping [cmH$_2$O/l], H: respiratory tissue elastance [cmH$_2$O/l], EELV: end-expiratory lung volume [ml].

		Sprague Dawley		Wistar	
		**female**	**male**	**female**	**male**
**PEEP0**	**Raw**	64.71 (35.29 – 126.99)	84.24 (30.97 – 222.91)	56.80 (31.71 – 165.05)	72.58 (26.16 – 181.37)
	**G**	939.41 (621.24 – 1619.09)	1073.72 (620.07 – 1991.47)	972.53 (666.39 – 2336.87)	1025.25 (610.54 – 2978.78)
	**H**	4045.41 (2745.52 – 7378.49)	4695.37 (2124.42 – 14492.82)	4133.12 (2181.16 – 14595.07)	4493.68 (2337.79 – 8635.15)
	**EELV**	3.48 (1.13 – 6.80)	4.12 (1.63 – 10.98)	3.27 (2.19 – 6.60)	3.67 (1.98 – 8.99)
**PEEP1**	**Raw**	54.07 (30.55 – 104.05)	62.64 (30.15 – 157.05)	46.00 (23.97 – 86.31)	60.11 (28.22 – 152.97)
	**G**	810.07 (573.63 – 1301.01)	919.91 (569.49 – 1748.74)	789.55 (595.89 – 1295.93)	867.30 (504.14 – 1764.08)
	**H**	3467.47 (2177.42 – 6414.10)	3805.83 (1828.55 – 9978.47)	3324.82 (1734.46 – 6593.67)	3782.82 (1871.62 – 6846.29)
	**EELV**	4.61 (1.63 – 9.72)	5.55 (2.49 – 15.18)	4.25 (3.17 – 8.85)	4.56 (2.34 – 10.91)
**PEEP2**	**Raw**	44.35 (24.44 – 80.48)	51.96 (22.81 – 105.26)	41.52 (27.00 – 78.26)	48.31 (23.62 – 113.55)
	**G**	734.08 (532.91 – 1242.73)	805.43 (511.60 – 1467.39)	682.28 (523.89 – 1096.18)	793.86 (442.43 – 1540.78)
	**H**	2957.49 (1648.31 – 5483.48)	3198.71 (1500.05 – 8312.81)	2898.11 (1513.42 – 5932.97)	3108.22 (1539.22 – 5681.22)
	**EELV**	5.28 (2.09 – 10.48)	6.71 (2.94 – 15.04)	4.97 (3.58 – 10.91)	5.39 (2.67 – 12.79)
**PEEP3**	**Raw**	39.06 (23.07 – 82.02)	43.74 (20.90 – 107.70)	37.07 (19.57 – 61.99)	43.92 (21.75 – 97.33)
	**G**	679.01 (495.41 – 996.63)	711.23 (461.44 – 1302.53)	624.05 (472.07 – 1037.36)	711.71 (410.44 – 1194.59)
	**H**	2460.08 (1380.25 – 4230.85)	2438.27 (1109.75 – 6989.33)	2390.54 (1294.01 – 5260.02)	2627.67 (1279.36 – 4481.54)
	**EELV**	6.25 (3.23 – 11.67)	7.97 (3.55 – 15.69)	5.78 (3.81 – 12.66)	6.64 (3.01 – 15.42)
**PEEP4**	**Raw**	32.56 (19.61 – 62.63)	38.31 (15.91 – 91.06)	33.46 (16.46 – 55.27)	38.22 (18.45 – 86.45)
	**G**	637.93 (472.20 – 926.48)	614.56 (431.46 – 1170.01)	589.23 (407.34 – 1009.85)	670.33 (396.07 – 1006.37)
	**H**	1995.69 (1200.39 – 3577.31)	1892.48 (1004.74 – 5590.10)	1873.02 (1045.59 – 4695.79)	2260.93 (1074.36 – 4489.53)
	**EELV**	7.10 (3.75 – 12.92)	9.07 (4.14 – 16.45)	7.06 (4.25 – 15.01)	7.63 (3.26 – 14.92)
**PEEP6**	**Raw**	25.74 (12.75 – 55.37)	27.32 (8.00 – 64.23)	25.06 (13.41 – 43.18)	27.72 (11.50 – 63.04)
	**G**	586.01 (447.07 – 827.68)	566.81 (413.27 – 1092.18)	539.06 (406.95 – 863.81)	607.38 (389.65 – 841.46)
	**H**	1554.86 (1121.84 – 3707.93)	1529.09 (944.00 – 4149.68)	1542.75 (926.66 – 3201.93)	1672.75 (936.04 – 3627.45)
	**EELV**	9.27 (4.17 – 14.34)	11.45 (5.79 – 20.27)	8.70 (4.75 – 16.34)	10.16 (3.86 – 16.69)

Model summaries

Below are the complete summaries of the final GAMLSS models used in the study, presented as direct copies of the R *summary()* output for transparency and reproducibility. The syntax for each model call is also included. Each model is presented on a separate page.

Model for Raw:

```
model_Raw <- gamlss(
  log(Raw) ~ sqrt(mass) + peep + sex + strain,
  sigma.formula = ~ strain + peep,
  family = NO(),
  data = dataset
)
> summary(model_Raw)
******************************************************************
Family:  c("NO", "Normal")

Call:  gamlss(formula = log(Raw) ~ sqrt(mass) + peep + sex + strain,
sigma.formula = ~strain + peep, family = NO(), data = dataset)

Fitting method: RS()

------------------------------------------------------------------
Mu link function:  identity
Mu Coefficients:
             Estimate Std. Error t value Pr(>|t|)
(Intercept)   5.36400    0.04937 108.657  < 2e-16 ***
sqrt(mass)   -0.07261    0.00265 -27.404  < 2e-16 ***
peep1        -0.20376    0.02778  -7.336 4.32e-13 ***
peep2        -0.36403    0.02764 -13.168  < 2e-16 ***
peep3        -0.51259    0.02824 -18.148  < 2e-16 ***
peep4        -0.65403    0.02968 -22.033  < 2e-16 ***
peep6        -0.96527    0.03150 -30.643  < 2e-16 ***
sexmale       0.24217    0.01689  14.339  < 2e-16 ***
strainwistar -0.07227    0.01605  -4.504 7.40e-06 ***
---
Signif. codes:  0 ‘***’ 0.001 ‘**’ 0.01 ‘*’ 0.05 ‘.’ 0.1 ‘ ’ 1

------------------------------------------------------------------
Sigma link function:  log
Sigma Coefficients:
             Estimate Std. Error t value Pr(>|t|)
(Intercept)  -1.17594    0.05734 -20.510  < 2e-16 ***
strainwistar -0.12428    0.04346  -2.859  0.00433 **
peep1        -0.19736    0.07406  -2.665  0.00782 **
peep2        -0.21221    0.07458  -2.845  0.00452 **
peep3        -0.16229    0.07495  -2.165  0.03058 *
peep4        -0.05226    0.07479  -0.699  0.48480
peep6         0.06301    0.07459   0.845  0.39840
---
Signif. codes:  0 ‘***’ 0.001 ‘**’ 0.01 ‘*’ 0.05 ‘.’ 0.1 ‘ ’ 1

------------------------------------------------------------------
No. of observations in the fit:  1098
Degrees of Freedom for the fit:  16
      Residual Deg. of Freedom:  1082
                      at cycle:  3

Global Deviance:     202.9776
            AIC:     234.9776
            SBC:     314.9976
******************************************************************
```

Model for G:

```
model_G <- gamlss(
    log(G) ~ sqrt(mass) + peep + strain + sex,
    sigma.formula = ~ sex + peep,
    family = NO(),
    data = dataset
)
> summary(model_G)
******************************************************************
Family:  c("NO", "Normal")

Call:  gamlss(formula = log(G) ~ sqrt(mass) + peep + strain + sex,
sigma.formula = ~sex + peep, family = NO(),data = dataset)

Fitting method: RS()

------------------------------------------------------------------
Mu link function:  identity
Mu Coefficients:
              Estimate Std. Error t value Pr(>|t|)
(Intercept)   7.875943   0.025792 305.363  < 2e-16 ***
sqrt(mass)   -0.060221   0.001369 -43.984  < 2e-16 ***
peep1        -0.172551   0.015661 -11.018  < 2e-16 ***
peep2        -0.279619   0.015438 -18.112  < 2e-16 ***
peep3        -0.368556   0.015753 -23.395  < 2e-16 ***
peep4        -0.452092   0.015598 -28.984  < 2e-16 ***
peep6        -0.537163   0.015291 -35.128  < 2e-16 ***
strainwistar -0.057167   0.008147  -7.017 3.99e-12 ***
sexmale       0.168878   0.008412  20.075  < 2e-16 ***
---
Signif. codes:  0 ‘***’ 0.001 ‘**’ 0.01 ‘*’ 0.05 ‘.’ 0.1 ‘ ’ 1

------------------------------------------------------------------
Sigma link function:  log
Sigma Coefficients:
            Estimate Std. Error t value Pr(>|t|)
(Intercept) -1.85751    0.05721 -32.467  < 2e-16 ***
sexmale      0.12800    0.04454   2.874 0.004134 **
peep1       -0.25486    0.07402  -3.443 0.000597 ***
peep2       -0.29300    0.07399  -3.960 7.99e-05 ***
peep3       -0.23964    0.07411  -3.233 0.001260 **
peep4       -0.26472    0.07417  -3.569 0.000374 ***
peep6       -0.32089    0.07477  -4.292 1.93e-05 ***
---
Signif. codes:  0 ‘***’ 0.001 ‘**’ 0.01 ‘*’ 0.05 ‘.’ 0.1 ‘ ’ 1

------------------------------------------------------------------
No. of observations in the fit:  1098
Degrees of Freedom for the fit:  16
      Residual Deg. of Freedom:  1082
                      at cycle:  3

Global Deviance:     -1302.832
            AIC:     -1270.832
            SBC:     -1190.813
******************************************************************
```

Model for H:

```
model_H <- gamlss(
  log(H) ~ sqrt(mass) + peep + strain + sex,
  sigma.formula = ~ mass + sex,
  family = NO(),
  data = dataset
)
> summary(model_H)
******************************************************************
Family:  c("NO", "Normal")

Call:  gamlss(formula = log(H) ~ sqrt(mass) + peep + strain + sex,
sigma.formula = ~mass + sex, family = NO(),data = dataset)

Fitting method: RS()

------------------------------------------------------------------
Mu link function:  identity
Mu Coefficients:
              Estimate Std. Error t value Pr(>|t|)
(Intercept)   9.986231   0.037823 264.026   <2e-16 ***
sqrt(mass)   -0.099475   0.002168 -45.879   <2e-16 ***
peep1        -0.207773   0.018898 -10.995   <2e-16 ***
peep2        -0.392985   0.018905 -20.787   <2e-16 ***
peep3        -0.586291   0.018919 -30.989   <2e-16 ***
peep4        -0.760921   0.018903 -40.254   <2e-16 ***
peep6        -0.984473   0.018965 -51.910   <2e-16 ***
strainwistar -0.056539   0.011180  -5.057    5e-07 ***
sexmale       0.201378   0.011450  17.588   <2e-16 ***
---
Signif. codes:  0 ‘***’ 0.001 ‘**’ 0.01 ‘*’ 0.05 ‘.’ 0.1 ‘ ’ 1

------------------------------------------------------------------
Sigma link function:  log
Sigma Coefficients:
              Estimate Std. Error t value Pr(>|t|)
(Intercept) -1.8937061  0.0538327 -35.178  < 2e-16 ***
mass         0.0007823  0.0001512   5.175 2.71e-07 ***
sexmale     -0.0768220  0.0464895  -1.652   0.0987 .
---
Signif. codes:  0 ‘***’ 0.001 ‘**’ 0.01 ‘*’ 0.05 ‘.’ 0.1 ‘ ’ 1

------------------------------------------------------------------
No. of observations in the fit:  1098
Degrees of Freedom for the fit:  12
      Residual Deg. of Freedom:  1086
                      at cycle:  6

Global Deviance:     -623.9423
            AIC:     -599.9423
            SBC:     -539.9273
******************************************************************
```

Model for EELV:

```
model_EELV <- gamlss(
  EELV ~ sqrt(mass) + peep + strain + sex,
  sigma.formula = ~ mass + peep + sex + strain,
  family = NO(),
  data = dataset
)
> summary(model_EELV)
******************************************************************
Family:  c("NO", "Normal")

Call:  gamlss(formula = EELV ~ sqrt(mass) + peep + strain + sex,
sigma.formula = ~mass + peep + sex + strain, family = NO(), data = dataset)

Fitting method: RS()

------------------------------------------------------------------
Mu link function:  identity
Mu Coefficients:
             Estimate Std. Error t value Pr(>|t|)
(Intercept)  -5.29284    0.28431 -18.616  < 2e-16 ***
sqrt(mass)    0.58809    0.01791  32.844  < 2e-16 ***
peep1         0.82506    0.09576   8.616  < 2e-16 ***
peep2         1.57536    0.10305  15.288  < 2e-16 ***
peep3         2.47960    0.11327  21.892  < 2e-16 ***
peep4         3.46782    0.12878  26.928  < 2e-16 ***
peep6         5.32320    0.17132  31.071  < 2e-16 ***
strainwistar -0.16721    0.06869  -2.434 0.015100 *
sexmale      -0.27947    0.07729  -3.616 0.000314 ***
---
Signif. codes:  0 ‘***’ 0.001 ‘**’ 0.01 ‘*’ 0.05 ‘.’ 0.1 ‘ ’ 1

------------------------------------------------------------------
Sigma link function:  log
Sigma Coefficients:
               Estimate Std. Error t value Pr(>|t|)
(Intercept)  -0.8784505  0.0874138 -10.049  < 2e-16 ***
mass          0.0041315  0.0001926  21.446  < 2e-16 ***
peep1         0.0226490  0.0775161   0.292 0.770206
peep2         0.1339900  0.0800003   1.675 0.094274 .
peep3         0.2762520  0.0820650   3.366 0.000791 ***
peep4         0.4572450  0.0828465   5.519 4.35e-08 ***
peep6         0.7014721  0.0886178   7.916 6.54e-15 ***
sexmale      -0.4110535  0.0486991  -8.441  < 2e-16 ***
strainwistar -0.1394502  0.0457932  -3.045 0.002386 **
---
Signif. codes:  0 ‘***’ 0.001 ‘**’ 0.01 ‘*’ 0.05 ‘.’ 0.1 ‘ ’ 1

------------------------------------------------------------------
No. of observations in the fit:  1012
Degrees of Freedom for the fit:  18
      Residual Deg. of Freedom:  994
                      at cycle:  7

Global Deviance:     3488.327
            AIC:     3524.327
            SBC:     3612.882
******************************************************************
```

Residual diagnostics

Supplementary Figure 1: Diagnostic plots for the fitted model on Raw: Quantile residuals against fitted values (top left), quantile residuals against index values (top right), density plot of residuals with normal distribution for reference (dashed line, bottom left), Q-Q plot with trend line indicated (bottom right).

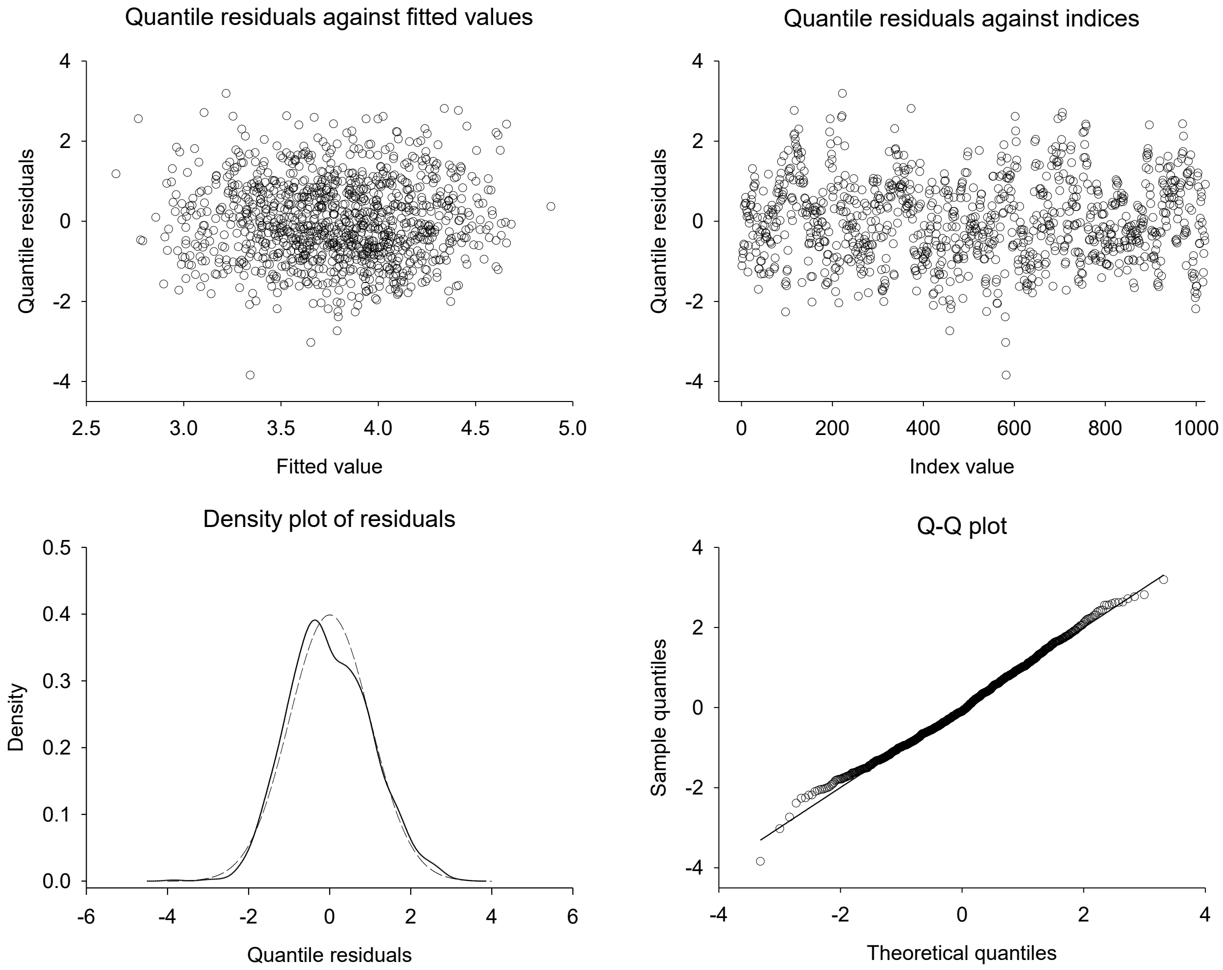

Supplementary Figure 2: Diagnostic plots for the fitted model on G: Quantile residuals against fitted values (top left), quantile residuals against index values (top right), density plot of residuals with normal distribution for reference (dashed line, bottom left), Q-Q plot with trend line indicated (bottom right).

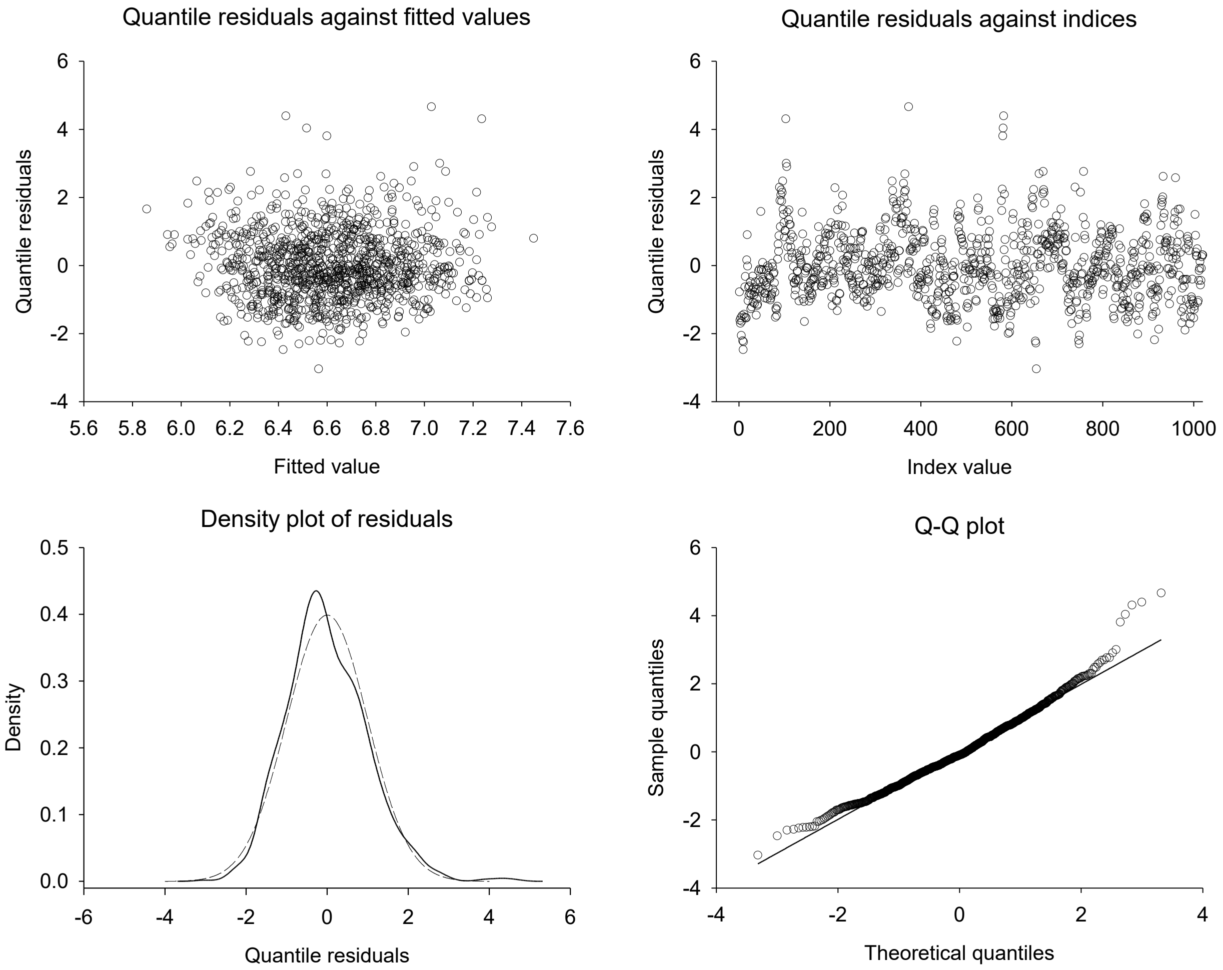

Supplementary Figure 3: Diagnostic plots for the fitted model on H: Quantile residuals against fitted values (top left), quantile residuals against index values (top right), density plot of residuals with normal distribution for reference (dashed line, bottom left), Q-Q plot with trend line indicated (bottom right).

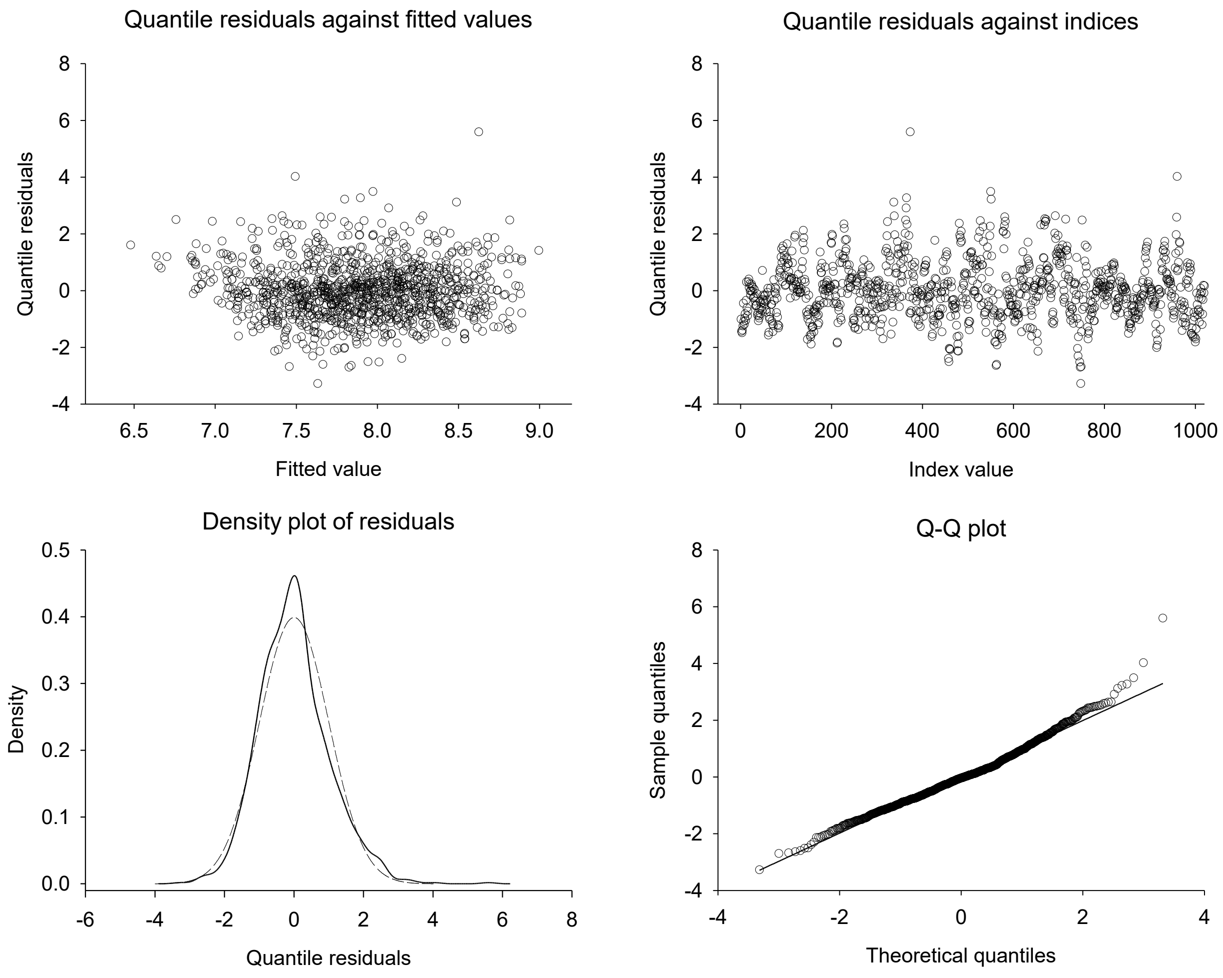

Supplementary Figure 4: Diagnostic plots for the fitted model on EELV: Quantile residuals against fitted values (top left), quantile residuals against index values (top right), density plot of residuals with normal distribution for reference (dashed line, bottom left), Q-Q plot with trend line indicated (bottom right).

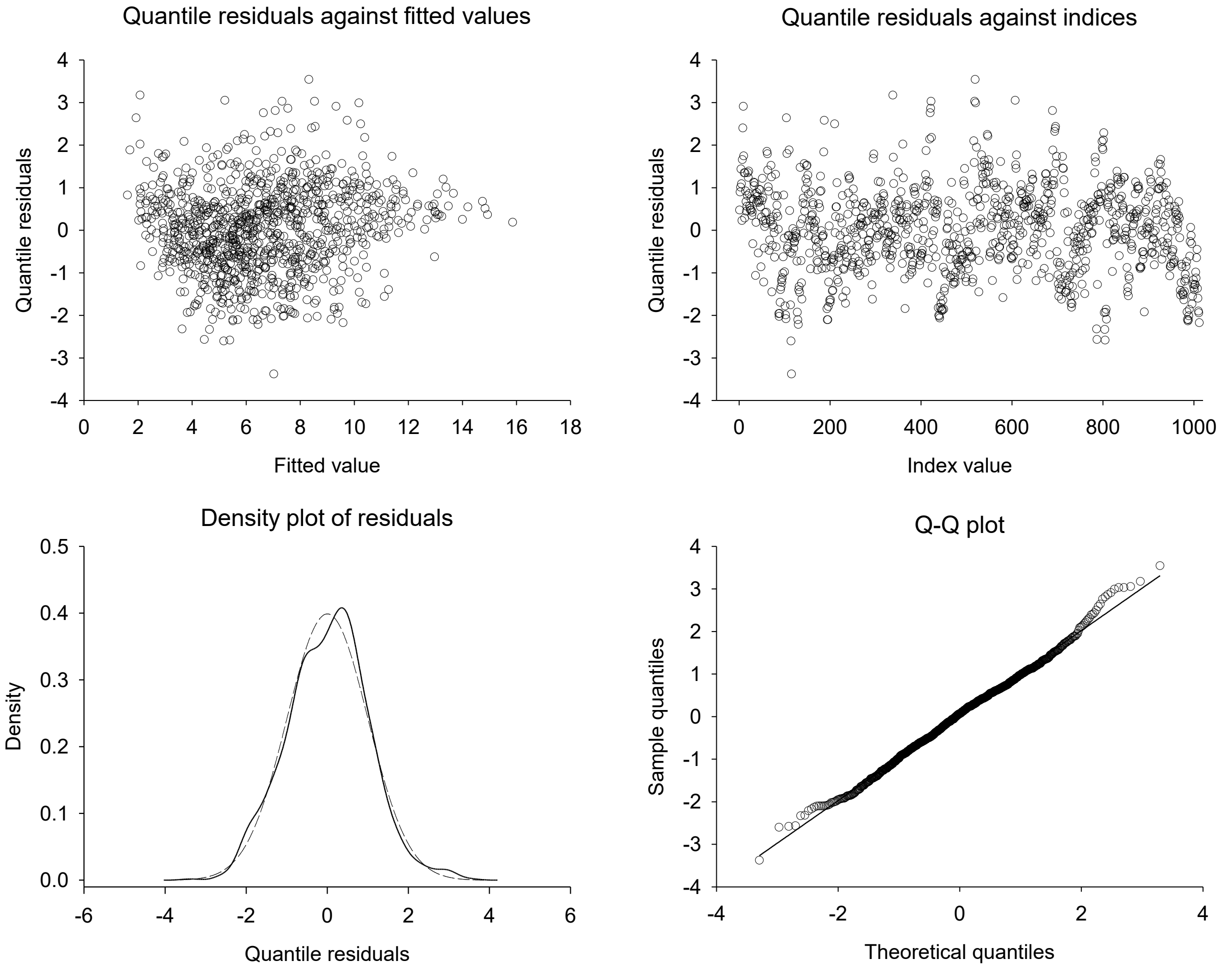

Applicability of the reference equations to new data

Supplementary Figure 5: *In vivo* validation of the reference framework using a bleomycin injury model. Absolute values of tissue elastance (H, left) show a clear separation between male and female animals (female with red, male with blue symbols), complicating direct comparison because absolute values are strongly influenced by body mass, which differed between males and females. When expressed as z-scores relative to sex- and body-mass-adjusted reference equations (right), this separation disappears, and the majority of bleomycin-treated animals exceed the upper limit of normal (ULN, defined as 95th percentile of the reference distribution, z = +1.645; shaded area). In contrast, all but one control animal remain within the expected healthy range. This example illustrates the framework's ability to harmonise measurements across sexes and body sizes, and its potential utility in preclinical study design.
n=6 for control (3 male, 3 female), n=11 for bleomycin (5 male, 6 female). All rats were Sprague Dawley and ventilated at PEEP6. All data comes from an independent dataset from an ongoing study not yet published. Boxes represent median and interquartile range; whiskers indicate the 10th and 90th percentiles; individual data points are shown.

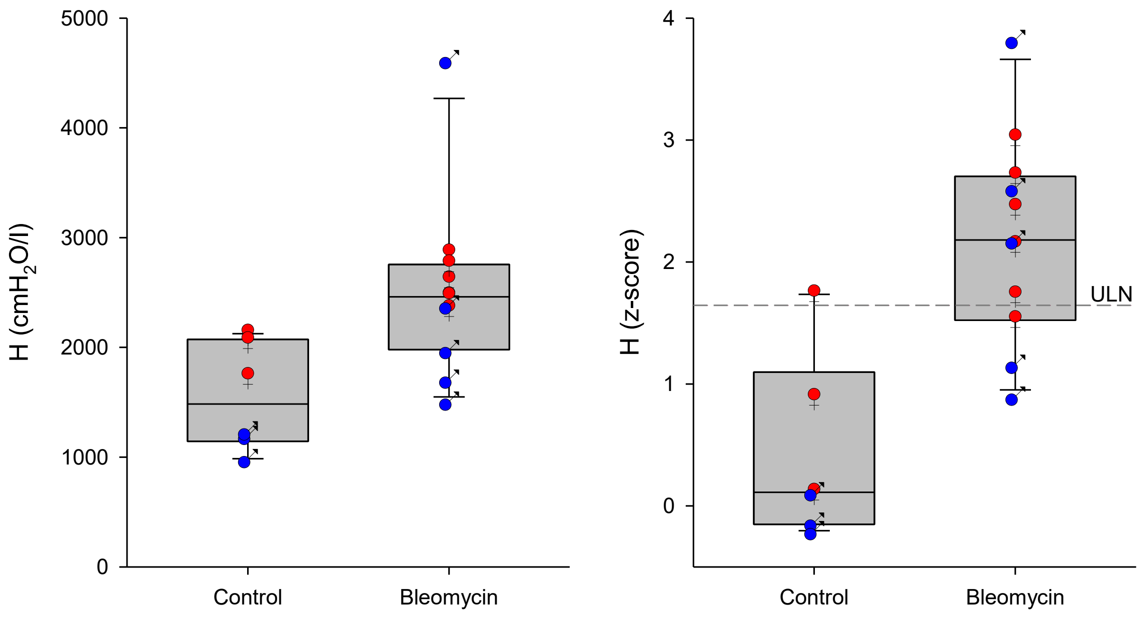

Effect sizes of the models

Supplementary Table 2: Effect sizes of strain and sex across the outcome variables derived from the GAMLSS models. Effect sizes are reported on the original measurement scale. For Raw, G, and H, which were modelled using a log-transformed response, effect sizes are multiplicative, and are expressed as percent change, calculated as $\%\Delta = \left(e^{\beta} - 1\right) \cdot 100\%$, where β is the corresponding regression coefficient for strain or sex. For EELV, which was modelled on the original scale using and identity link, percent change was estimated using a reference animal (Sprague-Dawley, female, PEEP0, mass of 260 g, representing the average rat) using the formula $\%\Delta_{EELV} = \frac{\beta_{\mu}}{\mu_{ref}} \cdot 100\%$.

To facilitate comparison of relative effect magnitude across outcome variables, standardized effect sizes are additionally provided. Standardized effect sizes (analogous to Cohen's d) are expressed in units of the model-implied standard deviation and were calculated by dividing the μ regression coefficient by the model-implied baseline standard deviation, obtained by exponentiating the intercept of the σ submodel. Standardized effect sizes are dimensionless and are intended solely for comparative interpretation across parameters; they do not represent absolute physiological changes. Standardized effect sizes can be classified as d(0.2)=small, d(0.5)=medium, d(0.8) = large, d(1.2)=very large [1].

Effects on variability were quantified from the σ submodels and are reported as percent change in the model-implied standard deviation, calculated as $\%\Delta_{\sigma} = \left(e^{\beta_{\sigma}} - 1\right) \cdot 100\%$, where β_{σ} is the corresponding regression coefficient from the σ submodel. Variability effect sizes are scale-free and describe proportional changes in dispersion rather than shifts in central tendency. If a factor was not present in the σ submodel, value is not calculated and is indicated by '---'.

Positive effect sizes for each calculation indicate higher values in males or Wistar rats relative to the reference categories (female or Sprague Dawley).

**Outcome**	**Factor**	**Effect size for mean value (original scale)**	**Standardized effect size [standard deviation unit]**	**Effect for variability (original scale)**
Raw	strain	-6.97 %	-0.23 (small)	-11.69 %
	sex	27.40 %	0.78 (large)	---
G	strain	-5.56 %	-0.37 (medium)	---
	sex	18.40 %	1.08 (very large)	13.66 %
H	strain	-5.50 %	-0.38 (medium)	---
	sex	22.31 %	1.34 (very large)	-7.39 %
EELV	strain	-3.99 %	-0.40 (medium)	-13.02 %
	sex	-6.67 %	-0.67 (medium-large)	-33.70 %

SUPPLEMENTARY NOTES

Detailed relationships between current results and other measurement modalities

Regarding respiratory mechanics, other methods, such as the multiple linear regression (MLR) approach, provide total respiratory elastance (Ers) and resistance (Rrs) using the equation of motion [2]. Rrs combines airway and tissue components, complicating direct comparison, but Raw, G and H can be used to estimate Rrs and Ers at a given frequency, based on the relationships $Rrs = Raw + G/(2\pi f)^{\alpha}$ and $Ers = H/(2\pi f)^{\alpha}$ (where f is the ventilation frequency and $\alpha = (2/\pi) \cdot \arctan(H/G)$, [3]). Methodological differences, including measurement over the full ventilation cycle in MLR vs. end-expiration in oscillometry should be still considered [4, 5]. Transfer impedance in conscious rodents has also been described [6], but differences between spontaneous and controlled ventilation preclude direct use of the present reference values [7].

In addition to other preclinical approaches to respiratory mechanics, clinical oscillometry represents a distinct measurement modality that samples overlapping mechanical domains of the respiratory system. While direct conversion between constant phase model parameters and oscillometric indices is not straightforward, conceptual correspondence between constant phase model parameters and oscillometric indices has been described previously, with central airway resistance reflected by high-frequency resistance (R20), peripheral heterogeneity and dissipation by low-frequency resistance differences (R5–R20), and tissue elastance by reactance-based measures (X5, AX) [8, 9, 10].

Regarding EELV, the reference equations may also be applied to values from inert gas washout[11] or imaging techniques [12], provided methodological differences are considered. Notably, plethysmography and imaging capture total intrathoracic gas volume, while inert gas washout reflects only alveolar compartments communicating with the airway opening.

Considerations about model selection

During our preliminary analyses besides the square root of body mass we also considered body mass without transformation and the square of body mass as a predictor, with the square root providing the best fits. Penalized splines were also considered for smoothing body mass, but cross-validation clearly showed that their inclusion resulted in poor generalizability and extreme standardized residuals at the distribution boundaries, despite their favorable GAIC values, suggesting overfitting in that case. In case of predictions for the standard deviation or the distributions, we decided to use body mass without any transformation to improve the transparency of the models.

With respect to the remaining candidate predictors, PEEP was considered a biologically relevant variable, as it directly determines end-expiratory lung volume at different pressure levels and, through this mechanism, also affects respiratory mechanics. Therefore, inclusion of PEEP in the model was physiologically justified. Strain and sex were included based on analogies to human reference value modelling, where sex and ethnicity are known to exert significant effects on respiratory parameters. Body mass, strain, sex, and PEEP were all evaluated as potential predictors for both the mean and variability of Raw, G, H, and EELV. However, predictors were retained in the final models only when they demonstrated a statistically significant contribution, as indicated by their model coefficients (as seen in Table 2 of the main manuscript).

Raw, G, and H were log-transformed to reduce skewness and improve model fit. In contrast, EELV exhibited substantially less skewness, and therefore log-transformation was not required. This decision was supported by residual diagnostics, which showed approximate normality for the EELV models without transformation. In addition, approximately linear relationships were observed between EELV and PEEP, as well as between EELV and body mass.

SUPPLEMENTARY REFERENCES

1. Sawilowsky SS. New Effect Size Rules of Thumb. *J Mod Appl Stat Meth* **8**, 597-599 (2009). DOI 10.22237/jmasm/1257035100
2. Bates JHT. The linear single-compartment model. In: *Lung Mechanics: An Inverse Modeling Approach*). Cambridge University Press (2009). Doi 10.1017/Cbo9780511627156.004
3. Hantos Z, Daroczy B, Suki B, Nagy S, Fredberg JJ. Input impedance and peripheral inhomogeneity of dog lungs. *J Appl Physiol* **72**, 168-178 (1992). 10.1152/jappl.1992.72.1.168
4. Similowski T, Bates JH. Two-compartment modelling of respiratory system mechanics at low frequencies: gas redistribution or tissue rheology? *Eur Respir J* **4**, 353-358 (1991)
5. Lundblad LK. Issues determining direct airways hyperresponsiveness in mice. *Front Physiol* **3**, 408 (2012). 10.3389/fphys.2012.00408
6. Oostveen E, Zwart A, Peslin R, Duvivier C. Respiratory transfer impedance and derived mechanical properties of conscious rats. *J Appl Physiol* **73**, 1598-1607 (1992). 10.1152/jappl.1992.73.4.1598
7. Bates JH, Irvin CG. Measuring lung function in mice: the phenotyping uncertainty principle. *J Appl Physiol* **94**, 1297-1306 (2003). 10.1152/japplphysiol.00706.2002
8. Hantos Z, Daroczy B, Suki B, Nagy S, Fredberg JJ. Input impedance and peripheral inhomogeneity of dog lungs. *J Appl Physiol (1985)* **72**, 168-178 (1992). 10.1152/jappl.1992.72.1.168
9. King GG*, et al.* Technical standards for respiratory oscillometry. *Eur Respir J* **55**, (2020). 10.1183/13993003.00753-2019
10. Kaczka DW, Dellaca RL. Oscillation mechanics of the respiratory system: applications to lung disease. *Crit Rev Biomed Eng* **39**, 337-359 (2011). 10.1615/critrevbiomedeng.v39.i4.60
11. Verbanck S, Gonzalez Mangado N, Peces-Barba G, Paiva M. Multiple-breath washout experiments in rat lungs. *J Appl Physiol (1985)* **71**, 847-854 (1991). 10.1152/jappl.1991.71.3.847
12. Cercos-Pita JL*, et al.* Lung tissue biomechanics imaged with synchrotron phase contrast microtomography in live rats. *Sci Rep* **12**, 5056 (2022). 10.1038/s41598-022-09052-9
