# Supplementary material for: Standardized lung function reference values in rats for translational respiratory research
Source: Commun Biol. 2026 Apr 24;9:626. doi: 10.1038/s42003-026-10123-0 (PMC13156265; doi:10.1038/s42003-026-10123-0)
Supplement: Supplementary file 2 — Description of Additional Supplementary Files [file 42003_2026_10123_MOESM2_ESM.docx]

**Description of Additional Supplementary File**

File name: Supplementary data 1
Description: Excel calculator for respiratory normal values in rats.

File name: Supplementary data 2
Description: Compiled R models and an example script to allow the use of the published results in R.
