# Supplementary material for: Standardized lung function reference values in rats for translational respiratory research
Source: Commun Biol. 2026 Apr 24;9:626. doi: 10.1038/s42003-026-10123-0 (PMC13156265; doi:10.1038/s42003-026-10123-0)
Supplement: Supplementary file 5 — Reporting Summary [file 42003_2026_10123_MOESM5_ESM.pdf]

nature portfolio

Corresponding author(s): Gergely H. Fodor

Last updated by author(s): Feb 6, 2026

Reporting Summary

Nature Portfolio wishes to improve the reproducibility of the work that we publish. This form provides structure for consistency and transparency in reporting. For further information on Nature Portfolio policies, see our Editorial Policies and the Editorial Policy Checklist.

Statistics

For all statistical analyses, confirm that the following items are present in the figure legend, table legend, main text, or Methods section.

n/a	Confirmed	
☐	☒	The exact sample size (*n*) for each experimental group/condition, given as a discrete number and unit of measurement
☐	☒	A statement on whether measurements were taken from distinct samples or whether the same sample was measured repeatedly
☐	☒	The statistical test(s) used AND whether they are one- or two-sided<br>*Only common tests should be described solely by name; describe more complex techniques in the Methods section.*
☐	☒	A description of all covariates tested
☐	☒	A description of any assumptions or corrections, such as tests of normality and adjustment for multiple comparisons
☐	☒	A full description of the statistical parameters including central tendency (e.g. means) or other basic estimates (e.g. regression coefficient) AND variation (e.g. standard deviation) or associated estimates of uncertainty (e.g. confidence intervals)
☐	☒	For null hypothesis testing, the test statistic (e.g. *F*, *t*, *r*) with confidence intervals, effect sizes, degrees of freedom and *P* value noted<br>*Give P values as exact values whenever suitable.*
☒	☐	For Bayesian analysis, information on the choice of priors and Markov chain Monte Carlo settings
☐	☒	For hierarchical and complex designs, identification of the appropriate level for tests and full reporting of outcomes
☐	☒	Estimates of effect sizes (e.g. Cohen's *d*, Pearson's *r*), indicating how they were calculated

Our web collection on statistics for biologists contains articles on many of the points above.

Software and code

Policy information about availability of computer code

Data collection	Data was collected by an in-house developed data measurement system for forced oscillation measurements.
Data analysis	Data was processed in Excel, then analysed in R (version 4.5.1), using the gamlss package (version 5.4-22).<br>An Excel calculator was created and compiled R models are also distributed with sample code. Both are available at Github at https://github.com/fodorgergely/reform-rat-lung-function

For manuscripts utilizing custom algorithms or software that are central to the research but not yet described in published literature, software must be made available to editors and reviewers. We strongly encourage code deposition in a community repository (e.g. GitHub). See the Nature Portfolio guidelines for submitting code & software for further information.

Data

Policy information about availability of data

All manuscripts must include a data availability statement. This statement should provide the following information, where applicable:

- Accession codes, unique identifiers, or web links for publicly available datasets
- A description of any restrictions on data availability
- For clinical datasets or third party data, please ensure that the statement adheres to our policy

ompiled R model objects, an Excel calculator, and custom R scripts for computing predicted values, percentiles (5th and 95th), and z-scores are provided as Supplementary Data and are also available at https://github.com/fodorgergely/reform-rat-lung-function 37. Percentiles are derived from the reference distribution

under the assumption of normal residuals (e.g. the 95th percentile corresponds to z = +1.645), which showed good agreement with observed data. The repository contains the most recent version of the models, scripts for model fitting and application, and full usage documentation. Additional data supporting the findings of this study are available from an institutional data repository with DOI indicated in the manuscript.

Research involving human participants, their data, or biological material

Policy information about studies with human participants or human data. See also policy information about sex, gender (identity/presentation), and sexual orientation and race, ethnicity and racism.

Reporting on sex and gender	No human participants are included.
Reporting on race, ethnicity, or other socially relevant groupings	No human participants are included.
Population characteristics	No human participants are included.
Recruitment	No human participants are included.
Ethics oversight	No human participants are included.

Note that full information on the approval of the study protocol must also be provided in the manuscript.

Field-specific reporting

Please select the one below that is the best fit for your research. If you are not sure, read the appropriate sections before making your selection.

☒ Life sciences ☐ Behavioural & social sciences ☐ Ecological, evolutionary & environmental sciences

For a reference copy of the document with all sections, see nature.com/documents/nr-reporting-summary-flat.pdf

Life sciences study design

All studies must disclose on these points even when the disclosure is negative.

Sample size	Since our data is descriptive in its nature, no sample size estimation was performed. Group sizes were chosen to allow for adequately powered regression analyses.
Data exclusions	Of 200 rats, 18 were excluded from the analysis, due to respiratory infection (3) premature loss (9), or incomplete data (6).
Replication	Respiratory measurements are in range with previous papers from our group and others, indicating successful replication.
Randomization	Animals were not randomized as rat strain and sex cannot be randomized. These factors were used as covariates of the statistical analysis. Positive end-expiratory pressure (PEEP) levels were applied in a strictly increasing order to standardize volume-history and to minimize carry-over effects from previous levels.
Blinding	Respiratory mechanical analyses were performed in a way where the analyser was blinded for rat strain and sex.

Reporting for specific materials, systems and methods

We require information from authors about some types of materials, experimental systems and methods used in many studies. Here, indicate whether each material, system or method listed is relevant to your study. If you are not sure if a list item applies to your research, read the appropriate section before selecting a response.

Materials & experimental systems

n/a	Involved in the study	
☒	☐	Antibodies
☒	☐	Eukaryotic cell lines
☒	☐	Palaeontology and archaeology
☐	☒	Animals and other organisms
☒	☐	Clinical data
☒	☐	Dual use research of concern
☒	☐	Plants

Methods

n/a	Involved in the study	
☒	☐	ChIP-seq
☒	☐	Flow cytometry
☒	☐	MRI-based neuroimaging

Animals and other research organisms

Policy information about studies involving animals; ARRIVE guidelines recommended for reporting animal research, and Sex and Gender in Research

Laboratory animals	Healthy Sprague Dawley (CD® IGS Rat, Charles River, Germany) and Wistar (Wistar IGS Rat, Charles River, Germany) rats of both sexes were studied. Of 200 rats, 18 were excluded from the analysis, due to respiratory infection (3) premature loss (9), or incomplete data (6). After all the exclusions, we included 52 male Sprague Dawley (median: 317 g, min-max: 170–750 g), 46 female Sprague Dawley (258 g, 164–376 g), 53 male Wistar (310 g, 156–530 g), and 31 female Wistar rats (260 g, 162–366 g) in the final analyses.
Wild animals	No wild animals have been used.
Reporting on sex	Rats from both sexes were used with data reported for both sexes separately as sex was a significant factor.
Field-collected samples	No field-collected samples were used.
Ethics oversight	This experimental protocol was approved by the National Food Chain Safety and Animal Health Directorate of Csongrád-Csanád County, Hungary (no. XXXII./2110/2019) on 16 December 2019. All procedures were carried out according to the guidelines of the Scientific Committee of Animal Experimentation of the Hungarian Academy of Sciences (updated Law and Regulations on Animal Protection: 40/2013 [II. 14.], the Government of Hungary) and in compliance with the ARRIVE and NIH guidelines.

Note that full information on the approval of the study protocol must also be provided in the manuscript.

Plants

Seed stocks	No plants were used.
Novel plant genotypes	No plants were used.
Authentication	No plants were used.
